# Systematic review and meta‐analysis of the pelvic organ prolapse and vaginal prolapse among the global population

**DOI:** 10.1002/bco2.464

**Published:** 2024-12-10

**Authors:** Tharanga Mudalige, Vindya Pathiraja, Gayathri Delanerolle, Heitor Cavalini, Shuqi Wu, Julie Taylor, Om Kurmi, Kathryn Elliot, Sharron Hinchliff, Carol Atkinson, Kristina Potocnik, Paula Briggs, Lucky Saraswat, Helen Felicity Kemp, George Eleje, Toh Teck Hock, Cristina Laguna Benetti‐Pinto, Irfan Muhammad, Rabia Kareem, Yassine Bouchareb, Peter Phiri, Ruishu Zhang, Yunfei Weng, Ieera Aggarwal, Jian Qing Shi, Ashish Shetty, Ian Litchfield, Nirmala Rathnayake, Sohier Elneil

**Affiliations:** ^1^ University of Ruhuna Matara Sri Lanka; ^2^ Southern Health NHS Foundation Trust Southampton UK; ^3^ University of Birmingham Birmingham UK; ^4^ Southern University of Science and Technology Shenzhen China; ^5^ University of Coventry Coventry UK; ^6^ University of Sheffield Sheffield UK; ^7^ Manchester Metropolitan University Manchester UK; ^8^ University of Edinburgh Edinburgh Scotland UK; ^9^ Department of Women's and Children's Health University of Liverpool Liverpool UK; ^10^ Liverpool Women's Hospital Foundation NHS Trust Liverpool UK; ^11^ University of Aberdeen Aberdeen Scotland UK; ^12^ No affiliation; ^13^ Nnamdi Azikiwe University Nnewi Nigeria; ^14^ Sibu Hospital Sibu Sarawak Malaysia; ^15^ University of Campinas, UNICAMP Campinas Brazil; ^16^ Peshawar Medical College Riphah International University Islamabad Pakistan; ^17^ Sultan Qaboos University Muscat Oman; ^18^ KK Women's and Children's Hospital Singapore; ^19^ National Centre for Applied Mathematics Shenzhen China; ^20^ University of Southampton Southampton UK; ^21^ University College London Hospitals NHS Foundation Trust London UK; ^22^ Institute for Women's Health, Faculty of Population Health Sciences University College London London UK

**Keywords:** gynaecology, pelvic organ prolapse, urology, wellbeing, women's health

## Abstract

**Background:**

Pelvic organ prolapse (POP) occurs when one or more pelvic organs (uterus, bowel, bladder or top of the vagina) descend from their normal position and bulge into the vagina. Symptoms include pelvic discomfort, fullness, and changes in bladder or bowel function. Treatment ranges from conservative approaches to surgery, depending on symptom severity. Surgical methods include vaginal wall repair, with or without hysterectomy, or via laparoscopic, robotic or open techniques. Common complications include bleeding, infection, and urinary or bowel dysfunction.

**Methods:**

A systematic review was conducted, and a protocol was registered with PROSPERO (CRD42022346051). Publications from 30 April 1980 to 30 April 2023 were retrieved from multiple databases. Data were analysed using random‐effects and common‐effects models with subgroup and sensitivity analyses.

**Findings:**

Forty‐four studies met the inclusion criteria, with 29 studies used for meta‐analysis of vaginal prolapse surgery outcomes. Sixteen studies focused on patients who had undergone hysterectomy alongside prolapse repair.

**Interpretation:**

Patients who underwent vaginal prolapse surgery with hysterectomy experienced higher operative and postoperative complication rates than those without hysterectomy. Increased risks included hospital readmission, POP recurrence and re‐operation. The review highlighted a lack of diversity in terms of ethnicity, age and comorbidity status, which are essential to fully understanding the impact of POP. Future research should focus on these underrepresented factors.

## 1 INTRODUCTION

The International Continence Society and International Urogynaecology Association define pelvic organ prolapse (POP)^1^ as the descent of one or more of the anterior vaginal wall, posterior vaginal wall, the uterus (cervix) or the apex of the vagina (vaginal vault or cuff scar after hysterectomy)^1^ . The presence of any such sign should be correlated with relevant POP symptoms. Most commonly, this correlation would occur at the level of the hymen or beyond. POP can happen because of weakening of the muscles and ligaments that support the pelvic organs, often as a result of childbirth, aging, chronic coughing, obesity or pelvic surgery. A prolapse is not life‐threatening, but it can cause pain and discomfort. Women experiencing vaginal prolapse may notice a bulge or protrusion outside the vaginal opening, pelvic discomfort, a sense of fullness and changes in bladder and bowel function, often resulting in a rectocele or cystocele. When multiple symptoms present, the prolapse becomes classified as a complex prolapse.^1^, ^2^, ^3^ Complex vaginal prolapse refers to cases where multiple pelvic organs prolapse simultaneously, often leading to more noticeable symptoms.^4^ Mild cases of vaginal prolapse may not cause significant discomfort or require immediate treatment. They can be managed using conservative management strategies, including lifestyle modifications, pelvic floor exercises and vaginal pessaries that are often sufficient to alleviate symptoms.^5^, ^6^

Persistent symptoms, such as pelvic pain, a sensation of pelvic heaviness or fullness, and changes in bladder and bowel function, may indicate the need for surgical treatment approaches.^7^ Minimally invasive surgical procedures, such as vaginal prolapse surgery of the anterior and posterior vaginal walls, are commonly used to repair and reposition the prolapsed organs. More complex procedures incorporating vaginal hysterectomy (VH), mesh or fascial augmented vaginal prolapse and/or abdominal prolapse surgery (performed open or using laparoscopy or robotic techniques), and/or concomitant continence surgery like colposuspension or autologous fascial sling may be necessary to achieve optimal results, particularly in complex cases of vaginal prolapse. However, the decision to undergo surgery, be it simple or complex, should be based on a thorough evaluation of the risks and benefits, as well as the patient's overall health and preferences.^8^

It is important for women experiencing symptoms of vaginal prolapse to consult with a healthcare professional for an accurate diagnosis and personalized treatment plan.^9^ A comprehensive approach that considers the severity of the prolapse, the presence of other medical conditions and the patient's individual needs and preferences is essential for achieving the best possible outcomes. Often, VH is undertaken as part of POP surgery. Within the wider context of gynaecology, hysterectomy stands as one of the most frequently performed surgical procedures in women, second only to caesarean delivery among non‐pregnancy‐related surgeries.^10^ The abdominal approach, though commonly employed, is associated with the highest complication rates, extended recovery periods and elevated costs.^11^ In contrast, laparoscopic hysterectomy, heralded as a potentially less invasive alternative to the abdominal method, has been hindered by slow skill development and has exhibited higher complication rates and costs compared with VH.^12^ Thus, VH, characterized by its minimal complications, reduced recovery burden and lower costs, emerges as a compelling candidate for the preferred approach.

The treatment of vaginal prolapse has been investigated in several randomized clinical trials, but a systematic overview of the topic is still lacking. We identify the features of vaginal prolapse, prevalence of vaginal prolapse among women who have had a hysterectomy versus those who have not and prevalence of vaginal prolapse among races and ethnicities in a systematic review and meta‐analysis.^12^ This has been combined with a network plot, thus utilizing the most reliable evidence coming from randomized controlled trials (RCTs). Literature reviews, commentaries and editorials in particular have focused on either complications or the quality of life of patients with POP. Therefore, we conducted an evidence synthesis of the current published literature to explore the key features of vaginal prolapse, including the prevalence and incidence among women of different races and ethnicities, as part of a consolidation of the available data to demonstrate existing knowledge and gaps in practice.

## 2 METHODS

A systematic methodology was developed to determine the prevalence and outcomes of vaginal prolapse among the global female population. A protocol was designed, peer reviewed and published on PROSPERO (CRD42022346051).

### 2.1 Aim

The primary aim was to report the prevalence of POP in women who underwent POP surgery with or without a hysterectomy. The secondary aims were to report complications in patients with POP who have undergone hysterectomy compared with those who had not and to report the prevalence of complications in patients who had undergone vaginal prolapse surgery.

### 2.2 Search strategy

**FIGURE 1:**
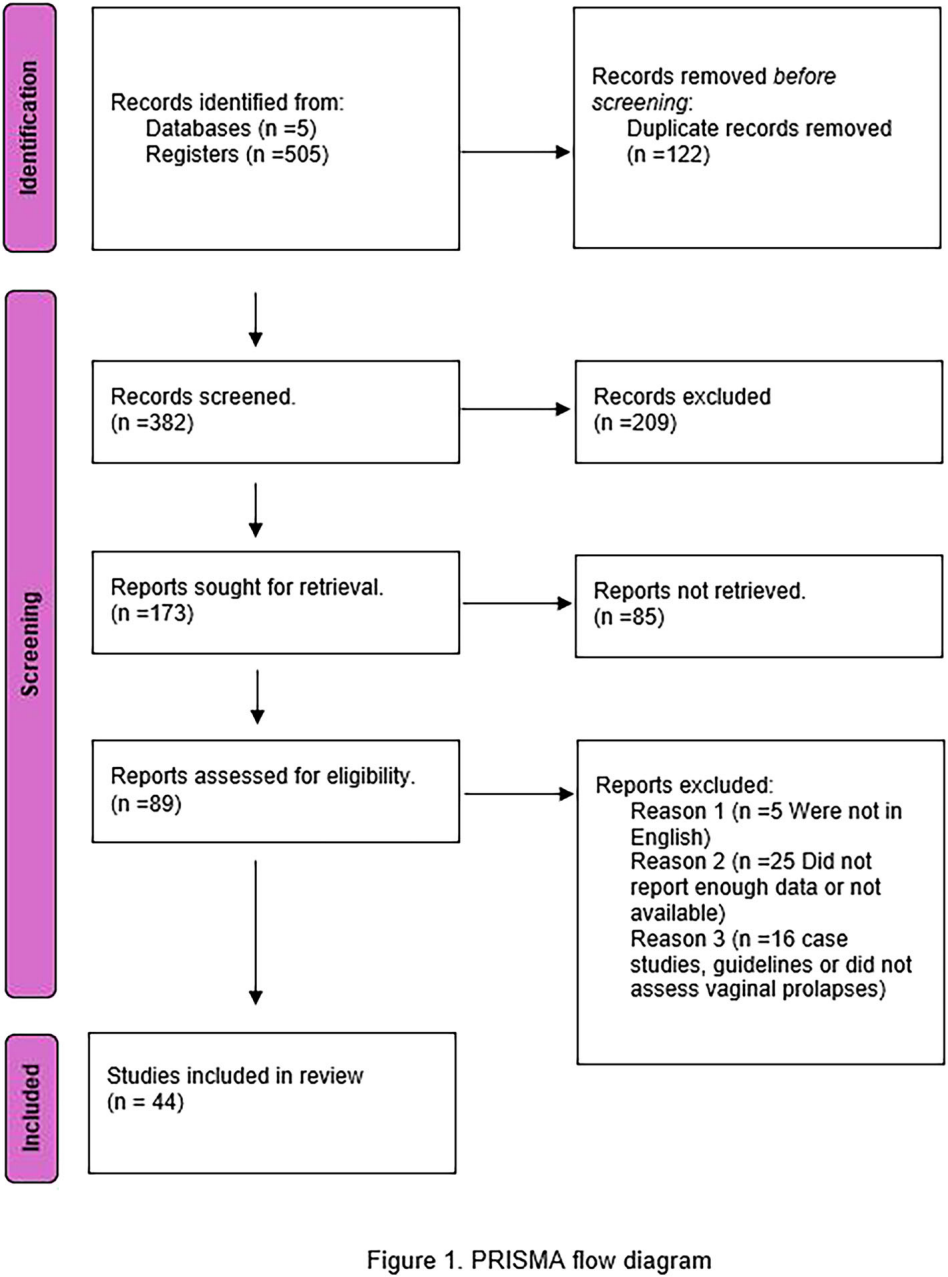
PRISMA flow diagram.

The search strategy comprised the use of multiple MeSH terms, including ethnicity; female; humans; hysterectomy; prevalence; race factors; racial groups; uterine prolapse; and women. The databases used included PubMed, Web of Science, ScienceDirect, The Cochrane Gynaecology and Fertility Group (CGF) Specialised Register of Controlled Trials, ProQuest, Embase and MEDLINE/OvidSP. Further details are provided in Figure 1.

### 2.3 Eligibility criteria

All studies with different study designs, including RCTs and/or cross‐sectional, qualitative and quantitative studies, were included. Studies published in English from the 30th of April 1980 to the 30th of April 2023 were included in this study.

### 2.4 Data extraction and synthesis

Data were extracted from studies that met the inclusion criteria. Pooled odds ratios (ORs) and 95% confidence intervals (CIs) were reported for overall and sub‐groups.

The data extraction process was documented using PRISMA (Figure 1). The data extraction and refinement processes were completed using Endnote and Microsoft Excel by six reviewers (TM, VP, HC, SW, YW and GD). An independent reviewer evaluated the dataset prior to the statistical analysis.

### 2.5 Statistical analysis plan

Studies included were categorized based on statistical and clinical characteristics. The pooled evidence was synthesized based on relative risks (RRs), OR, prevalence risks (PRs) and median, while mean differences (MDs) and their 95% CI were collated as part of the data synthesis. Key characteristics from the pooled data were tabulated and analysed as part of sensitivity and sub‐group analyses. These were also reported narratively using a thematic content analysis that included socio‐cultural factors, clinical and health outcomes, including surgical techniques used, procedures conducted such as hysterectomy, partial hysterectomy, oophorectomy or salpingectomy, and any patient reported outcomes.

### 2.6 Outcomes

Outcomes for the study included surgical and clinical outcomes, including complications among women with POP who have had a hysterectomy. We also explored complications included in the analysis, such as bladder injury, infections, recurrence of POP, readmission rates, reoperation rates, mesh complications in those who had mesh‐augmented surgery (such as extrusion or exposure into surrounding organs) and overall complications.

### 2.7 Risk of bias (quality assessment)

All literature identified and reported were appraised critically against the predefined variables. Independent reviewers indicated methodological quality and rigour. The Newcastle–Ottawa scale was used to determine the quality of the studies included within the meta‐analysis (Table S1b). This was furthered by the application of the refinement protocol (Figure 1), where all studies included in both meta‐analyses were evaluated against the eligibility criteria that demonstrated the scientific basis of the analysis conducted. The quality of the included cross‐sectional studies was measured using the modified Newcastle‐Ottawa Measurement Scale (NOS) specific for cross‐sectional studies. We rated the quality of the studies (good, fair and poor) by allocating each domain with stars in this manner.

- A good quality score was awarded 3 or 4 stars in selection, 1 or 2 in comparability and 2 or 3 stars in outcomes.
- A fair quality score was awarded 2 stars in selection, 1 or 2 stars in comparability and 2 or 3 stars in outcomes.
- A poor‐quality score was allocated 0 or 1 star(s) in selection, 0 stars in comparability and 0 or 1 star(s) in outcomes domain in line with the NOS guidelines.

Table 1 shows the sample characteristics.

**TABLE 1:** M1: prevalence of complications among women with POP that have had a hysterectomy. M2: prevalence of complications among women with POP that have not had a hysterectomy. M3: prevalence of complications among women with POP that have had and have not had a hysterectomy. M4: prevalence of complications among women with vaginal prolapse that have had a hysterectomy. Yes (Y)/No (N): not included in meta‐analysis.

Study ID	Author	Study type	Sample size	Exposure	Outcomes	Outcome measures	Included in the meta‐analysis Y/N
1	Elzaher M. A. et al.^22^	RCT	80	Either vaginal or abdominal hysterectomy for menorrhagia or other with uterine size <20 weeks, Gn RH‐a pre‐operatively for trial group (VH), prophylactic antibiotics, prophylactic anticoagulants	Uterine size, uterine bulk, histological uterine weight, post‐operative complications, blood loss, operating time, blood transfusions, pain at Day 0 and Day1	Ultrasound, estimated blood loss, visual analogue pain scale, clinical observation	N
2	Antosh D. D. et al.^13^	Prospective observational cohort study	64	Vaginal hysterectomy with bilateral salpingectomy. Benign surgical conditions such as prolapse, menorrhagia and fibroids	Proportion of successfully completed salpingectomies, operating time, blood loss, complications	Menopause Rating Scale, estimated blood loss, records of operating time and blood loss	M1
3	Arsene E. et al.^14^	Retrospective monocentric observational case series	27	Patients who underwent a medical or an immediate complication and reoperation following sacro‐colpopexy with mesh exposure (occurring intraoperatively or during the first hospital stay)	Surgical complications, reoperation	Medical records, over‐the‐phone follow‐up	M1
4	Aydin S. et al.^15^	Prospective cohort trial study	47 VALS, 32 SA	Vaginally assisted laparoscopic sacrocolpopexy (VALS), abdominal sacrocolpopexy (AS), pelvic organ prolapse	Operation times, anaesthesia times, estimated blood loss, middle‐term outcomes, perioperative and postoperative complications	Clinical records, complications, minimum 12‐month follow‐up	M3
5	Barboglio P.G. et al.^16^	Retrospective cohort study	127	Women undergoing robotic assisted laparoscopic sacrocolpopexy (RALS) with and without concomitant robotic assisted supra cervical hysterectomy, symptomatic pelvic organ prolapse.	Post‐operative complications, mesh extrusion, bowel injury re‐admission rate, wound infection, post‐operative hernia, recurrent prolapse	Subjective and objective follow‐up data, Baden–Walker (BW) preoperative POP grading and subjective data retrieved from self‐reported quality of life (QOL) validated questionnaires administered preoperatively and postoperatively. Pelvic Floor Distress Inventory‐20, Pelvic Floor Impact Questionnaire‐7 questionnaire. The PFIQ‐7 contains a urinary (UIQ), a colorectal (CRAIQ) and a prolapse (POPIQ) rate scale; the PFDI‐20 is comprised of 3 surveys assessing prolapse (POPDI), colorectal (CRADI) and the short version of the urinary distress inventory (UDI‐6).	×
6	Bogani G. et al.^23^	Retrospective cohort study	200	Hysterectomy for benign uterine disease except POP (VH or LH), BMI > 30 kg/m^2^	Outcomes between laparoscopic (LH) and vaginal (VH) hysterectomy operating time, estimated blood loss, complication development, hospital stay	Clinical records, follow‐up treatment	×
7	Bogani G. et al.^24^	Retrospective cohort study	80	VH or LH for older women >65 years, benign uterine condition (propensity matched)	Outcomes between laparoscopic (LH) and vaginal (VH) hysterectomy operating time, estimated blood loss, complication development, hospital stay	Baden–Walker grade, clinical records, readmission	×
8	Bojahr B. et al.^25^	Retrospective cohort study	310	Descensus with laparoscopic sacropexy with mesh attachment	Post‐OP examination (months), Descensus rezidiv/persistence, *n* (%), Cystocele, *n* (%), Rectocele, *n* (%), Preoperative Urinary Incontinence, *n* (%), Postoperative Urinary Incontinence	Peri‐operative examination and identification of complications, anatomical outcomes using clinical records	M1
9	Bradley M. S. et al.^26^	Retrospective cohort study	52	Robotic sacrocolpopexy, stage II, III, IV prolapse	Concomitant procedures *n* (%) Robotic supracervical hysterectomy 23 (44.0) Lysis of adhesions 21 (40.0) Midurethral sling 5 (9.6) Robotic salpingo‐oophorectomy 5 (9.6) Operative time, patient outcome, post‐operative complications	Retrospectively assessed clinical records	M1
10	Bretschneider C. E. et al.^27^	Retrospective cohort study	11 779	Laparoscopic colpopexy, intraperitoneal colpopexy, extraperitoneal colpopexy, colpoclesis, vaginectomy, anterior repair, posterior repair, and combined anterior–posterior repair, pelvic organ prolapses	No complication *N* = 10 372 Complication *N* = 1407 *p*‐value no reoperation *N* = 11 196 Reoperation *N* = 583 *p* no readmission *n* = 10 886 Readmission *n* = 893 *p*‐value	Clinical record evaluation for 1‐year re‐operation, 90‐day readmission, incidence of complications	M3
11	Bui C. et al.^58^	Prospective cohort study	101	Prolapse of grade greater than or equal to 2, laparoscopic promontofixation	Complications, patient satisfaction, persistant pelvic pain requiring removal of material, bladder injury, hematoma formation, vaginal erosion, operative time	POP‐Q classification, Quality of life was evaluated using the questionnaires Pelvic Floor Distress Inventory (PFDI 20), Pelvic Floor Impact Questionnaire (PISQ 7) and Pelvic Organ Prolaps/Urinary Incontinence Sexual Questionnaire (PISQ 12)	×
12	Cardenas‐Trowers O. et al.^59^	Retrospective cohort study	524	Women who underwent MISCP with LAVH, VH, LSCH and TLH	Including operative time, length of hospital stays, a composite outcome of 30‐day postoperative adverse events, readmission or reoperation	Clinical records evaluated for adverse incidents, 30‐day post‐operative adverse events and 90‐day re‐admissions	M1
13	Ceccaroni M. et al.^28^	Retrospective cohort study	8635	Total hysterectomy via LH, VH and AH	Eviscerated patients *n* % *p* Vaginal cuff dehiscence *n*%	30‐day post‐operative follow‐up for adverse event identification	×
14	Cengiz H. et al.^29^	RCT	49	Advanced uterine prolapse, VALH, VH + VVS	The main primary outcome measure was apical prolapse recurrence. Secondary results were duration of surgery, pain score, blood loss, postoperative hospital stay and quality of life scores related to prolapse.	POP quantification (POP‐Q) examination and validated questionnaires such as the International Consultation on Incontinence Questionnaire Vaginal Symptoms (IVIQ‐VS) survey, Urogenital Distress Inventory (UDI‐6), Incontinence Impact Questionnaire Short Form (IIQ‐7) and Patient Global Impression of Improvement (PGI‐I).	×
15	Chapman G. C. et al.^30^	Retrospective cohort study	3349 + 484	Patients who underwent USLS and total vaginal hysterectomy (TVH‐USLS), or USLS and total laparoscopic hysterectomy	Composite complication rate Urinary tract infection Superficial infection Serious complication rate Readmission Reoperation Blood transfusion Serious infectious complications Nonhome discharge Pneumonia Cystotomy Thromboembolism Cardiac complication Reintubation Death	30‐day post‐operative complication evaluation 90‐day readmission rate	M1
16	Chong W. et al.^31^	Retrospective cohort study	91 480	Pelvic organ prolapse	Venous thromboembolism (VTE), demographics, preclinical variables and route of surgery.	x	M3
17	Clancy A. A. et al.^32^	Retrospective cohort study	*n* = 57 233	Pelvic organ prolapse	Hospital readmission related to surgery within 30 days	x	M3
18	Costantini E. et al.^33^	Retrospective cohort study	179	x	x	x	×
19	Crouss T. et al.^34^	Retrospective cohort study	698	x	x	x	×
20	Dallas K. et al.^60^	Cohort study	12 189	x	x	x	×
21	Daniels S. et al.^35^	Retrospective cohort study	158	Uterovaginal prolapse	Primary outcome is the success rate according to the pelvic organ prolapse quantification (POP‐Q) system. Secondary measures included complication rates and patients identified as having Stages III–IV prolapse and their outcomes.	Clinical records to identify complications	M2
22	Dubinskaya A. et al.^36^	Retrospective cohort study	816	x	Complications, a composite variable including ≥1 transfusion, infection, readmission, reoperation, bowel obstruction/ileus, conversion to laparotomy, bowel/bladder injury or mesh complication. Logistic regression compared prolapse recurrence defined as retreatment (pessary/surgery) or postoperative POP‐Q points ≥ 0.	Clinical records to identify complications Readmission rate	M1
23	Fairchild et al.^37^	Qualitative	1557	Prolapse	Determine rates of concomitant procedures for POP in hysterectomies performed with POP as an indication, (2) identify factors associated with performance of a colpopexy at the time of hysterectomy for POP and (3) identify the influence of surgical complexity on perioperative complication rates.	x	M1
24	Fayyad A. et al.^38^	Qualitative	247	Hysterectomy vaginal prolapse	Postoperatively, patient symptoms, anatomical outcomes, mesh complications and patient global impression	Pre‐operative evaluation included symptoms' assessment using the Prolpase Quality of Life Questionnaire (P‐QOL) and objective assessment using the POP‐Q scores.	M1, M4
25	Fayyad A. M. et al.^39^	Qualitative	70	Uterine prolapse	General health Prolapse impact Role limitations Physical limitations Social limitations Personal relations Emotions Sleep Severity measures	Prolapse quality of life Questionnaire (P‐QOL) and underwent examination using pelvic organ prolapse quantification system (POP‐Q) pre‐ and post‐operatively.	M2
26	Fernandez H. et al.^61^	Qualitative	359	Benign uterine disease without prolapse or pelvic floor relaxation.	Changes in the rates of the type of hysterectomy over time	x	×
27	Ferreira H. et al.^40^	Qualitative	20	x	x	Women's demographic data and prolapse grade were evaluated preoperatively using the Pelvic Organ Prolapse Quantification score. Postoperative pain and surgical scar satisfaction were measured using the visual analogue pain scale and Patient and Observer Scar Assessment Questionnaire.	×
28	Freeman et al.^41^	Qualitative	30	Symptomatic and bothersome post‐hysterectomy vaginal vault prolapse	The primary outcomes were the quantitative description of point C (the apex/vault on the pelvic organ). Prolapse Quantification System [POP‐Q] [19] and the subjective Patient Global Impression of Improvement [PGI‐I] [20]	POP‐Q and the PGI‐I, morbidity and quality of life (P‐QOL).	M1
29	Gabriel I. et al.^42^	Qualitative	2158	Women undergoing hysterectomy across all indications (benign and malignant) between 2001 and 2008	Peri‐operative characteristics across hysterectomy routes, demographic data of women who underwent hysterectomy between 2001 and 2008 and were included versus excluded from analysis/incidence of prolapse after different modes of hysterectomy by type and grade of prolapse/survival analysis across three modes of hysterectomy with known prolapse status	x	M1
30	Gagnon L. H. et al.^43^	Qualitative	132 + 131	Women who underwent inpatient pelvic reconstructive surgery	Length of stay beyond the first postoperative day.	x	×
31	Giugale L. E. et al.^44^	Qualitative	654	Women undergoing uterovaginal prolapse repair	Composite prolapse recurrence (prolapse beyond the hymen or retreatment with pessary or surgery), secondary outcomes included mesh complications, time to recurrence and overall reoperation for either prolapse recurrence or mesh complication.	x	M1
32	Gutman R. E. et al.^45^	Qualitative	74 + 76	Uterovaginal prolapse	Baseline POP‐Q stage Stage 2 Stage 3 Stage 4	Pelvic organ prolapse quantification examination and validated questionnaires were collected at baseline and 12 months, including the Pelvic Floor Distress Inventory Short Form.	M2
33	Hertel H. et al.^46^	Qualitative	101	Uterine and vaginal vault prolapses	Grade II Grade III–IV Cystocele grade II Cystocele grade III–IV Rectocele grade II Rectocele grade III–IV Urinary stress incontinence	x	M1
34	Houlihan S. et al.^47^	Qualitative	206	x	Symptomatic recurrence prolapse, *n* POPDI‐6 score, median (IQR) ANY retreatment for recurrent prolapse, *n* (%) Pessary for recurrent prolapse, repeat surgery for recurrent prolapse Dyspareunia Difficulty with sexual activity† Stopped sexual activity† Treatment for painful intercourse	POPDI‐6, UDI‐6 PROMIS	M1
35	Illiano E. et al.^48^	Qualitative	136	Urogenital prolapse	Voiding symptoms Storage symptoms, stress urinary incontinence, urgency urinary incontinence Sexually active Sexual disturbance Constipation	The patients preoperatively completed the self‐administered Urinary Distress Inventory Short Form (UDI‐6) [14], the Incontinence Impact Questionnaire–Short Form (IIQ‐7) [15] for urinary symptoms and the Female Sexual Function Index Questionnaire (FSFI) for sexual dysfunction.	M3
36	Izett‐Kay M. L. et al.^49^	Qualitative	1121	Women who underwent laparoscopic mesh sacrohysteropexy	Patient‐reported mesh complications requiring the removal of the hysteropexy mesh. The use or expectant use of chronic pain services and the new diagnosis of a systemic autoimmune disorder. Further secondary outcomes included subsequent reoperation for POP and type of procedure, reoperation for SUI, Patient Global Impression of Improvement in prolapse symptoms (PGI‐I prolapse) and the ‘Friends and Family test’, asking whether participants would recommend the surgery if undergoing treatment for the same condition	x	M2
37	Joshi V. M. et al.^50^	Qualitative	194	Uterine prolapse in premenopausal women	Recurrent uterine prolapse Cystocele grade 2 or more 1 Rectocele Enterocele Cervical elongation Tape erosion into the bladder Wound morbidity	x	M2
					Bleeding		
38	Khan A. et al.^51^	Qualitative	794 + 176	x	Perioperative complications and early treatment failure	x	M1
39	Kotani Y. et al.^52^	Qualitative	138 + 30 + 66 + 68	x	Recurrence rate. Reoperation rate. Cumulative recurrence rate (%).	x	M3
40	Kow N. et al.^53^	Qualitative	102 + 95 + 28 + 15	Uterine‐sparing prolapse	x	x	M2
41	Kuhn A. et al.^54^	Qualitative	224	x	x	Participants completed a standardized physical examination and interview before randomization to surgical groups. Prolapse was staged using the pelvic organ prolapse quantification (POP‐Q) system, a standardized quantitative method of prolapse assessment	M1
42	Kupelian A. S. et al.^55^	Qualitative	110	x	x	Pelvic Organ Prolapse Quantification Scale.	M2
43	Lauretta A. et al.^56^	Qualitative	30	Rectal prolapse associated with genital prolapse	x	x	×

### 2.8 Data analysis

A random‐effects model or a fixed‐effect model with an inverse variance method was used for the meta‐analysis^17^, and the heterogeneity was assessed by $I^{2}$. Statistical heterogeneity was assessed using the $I^{2}$ statistic. Significant heterogeneity was defined as $I^{2}$ greater than 50% and a *p*‐value of Cochrane's Q less than 0.05. In the presence of high heterogeneity, the random effects model was employed, while a fixed‐effect model was used if there was weak or no heterogeneity existed. Subgroup analyses were performed according to the geographical location of the study and the type of surgery. Sensitivity analysis was used to evaluate the robustness of the results. Funnel plots were used to demonstrate publication bias.

## 3 RESULTS

A total of 44 studies were systematically included, although 15 were excluded from the meta‐analysis. Of the 15 studies, 3 lacked categorical data, 6 patients did not have POP and 3 were not available as full manuscripts. One study was also not published in English. Two studies did not distinguish between individuals who had a hysterectomy versus those who did not have a hysterectomy. Figure 1 indicates the process of screening studies included in the meta‐analysis.

To assess the severity of symptomatologies and quality of life, studies used a variety of clinically validated questionnaires, such as pelvic organ prolapse quantification (POP‐Q) questionnaire, Baden–Walker grading scale, visual analogue pain scale, Pelvic Floor Distress Inventory (PFDI 20), Pelvic Floor Impact Questionnaire (PISQ 7), Pelvic Organ Prolapse/Urinary Incontinence Sexual Questionnaire (PISQ 12), Prolapse Quality of Life Questionnaire (P‐QOL) and self‐reported quality of life questionnaire.

Of the 29 studies that were eligible, 16 were used to calculate the prevalence of complications among POP women that had a hysterectomy and 7 for those that had not; 6 studies were used to compare complications between POP women who had a hysterectomy and those that had not; and 1 study was used to report the prevalence of complications among women with vaginal prolapse that had had a hysterectomy. Fourteen studies were used for a sensitivity analysis using the geographical location.

### 3.1 Categorization

#### 3.1.1 Prevalence of complications among women with pelvic organ prolapse who underwent vaginal hysterectomy as part of POP management

A meta‐analysis was conducted using either a random‐effects model or a fixed‐effect model with 16 studies to examine complications among women with POP following a hysterectomy. The complications discussed in most studies were infection, complications following the use of mesh, bladder injury and reoperation rates.

**FIGURE 2:**
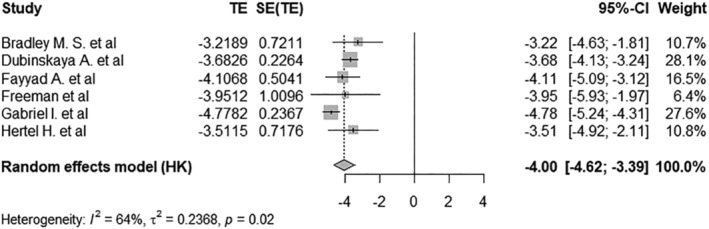
Forest plot showing the log odds of bladder injury among pelvic organ prolapse (POP) women that have had a hysterectomy*.*

**FIGURE 3:**
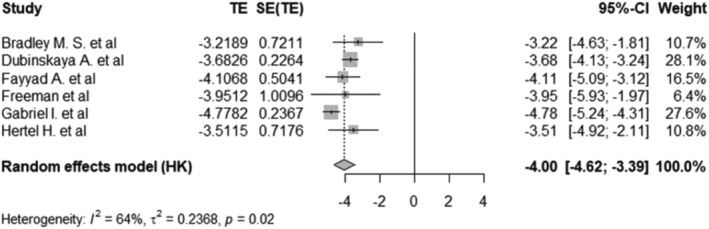
Forest plot showing the log odds of infections among pelvic organ prolapse (POP) women that have had a hysterectomy.

**FIGURE 4:**
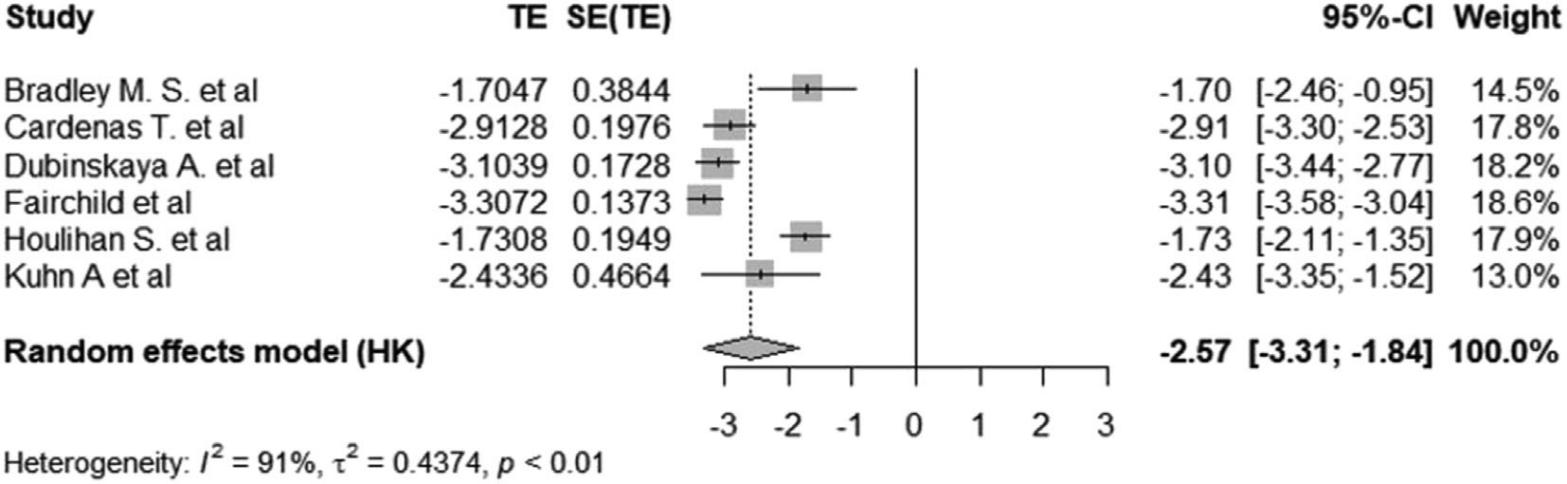
Forest plot showing the log odds of mesh complications among pelvic organ prolapse (POP) women that have had a hysterectomy.

The pooled prevalence of bladder injury among women with POP that had a hysterectomy, was 1.80% with a 95% CI of 0.98%–3.26%. Figure 2 shows the forest plot. Figure 3 demonstrates the pooled prevalence of infections was 7.11% with 95% CI of 3.52%–13.71% by using random‐effects model. The prevalence of mesh complications, calculated through the random‐effects model, was 4.88% (95% CI: 0.25%–50.1%), as evidenced in Figure 4. Four studies reported the prevalence of recurrence of POP in those that had had a hysterectomy in Figure S1. These studies reported the log‐odds. of −2.05 with a 95% CI of −2.40 to −1.70, which was equivalent to the prevalence of 11.41% with a 95% CI of 8.32%–15.45%. Three studies reported a prevalence of readmission of 3.49% (95% CI: 2.87%–4.23%) under the fixed‐effect model among POP women that have had a hysterectomy in Figure S2. Figure S3 illustrates the prevalence of reoperation rates under the random effects model was 3.59% (95% CI: 2.23%–5.79%).

Overall pooled prevalence of complications (other than recurrence, readmission and reoperation rates) among women with POP that had undergone hysterectomy was 10.53% (95% CI: 5.79%–18.39%) using a random effects model (Figure S4). Notably, the analysis revealed a high heterogeneity ($I^{2}$ = 97%) among the studies, attributed to variations in study type, covariates, assessment tools, ethnicities and other contributing factors.

These provided information on the prevalence of complications in women with POP after hysterectomy; however, it may need a further investigation into the factors contributing to heterogeneity among studies.

#### 3.1.2 Prevalence of complications among women with vaginal prolapse that have had a hysterectomy

Only one study reported the complications of vaginal prolapse surgery in patients who had undergone a hysterectomy prior to their current POP diagnosis and management. The prevalence of POP recurrence was 14.2%, the reoperation rate was 6.5%, bladder injury was 1.6%, mesh complications were 1.2% and overall complications were 4.0%.

### 3.2 Subgroup analysis

A subgroup analysis was conducted based on geographical regions correlated with the seven symptoms of bladder injury, infections, mesh complications, recurrence rates, readmission rates, reoperation rates and overall complications (other than recurrence, readmission and reoperation rates).

#### 3.2.1 Prevalence of complications among women who underwent pelvic organ prolapse surgery with hysterectomy in different geographical locations

Among patients with POP who had hysterectomies, studies in the Americas (mainly the United States) reported a higher prevalence of each symptom, followed by Europe and Australia. The prevalence of most symptoms was lower in the Americas and Australia. Due to the small number of subgroup studies, conclusions may be biased; therefore, we focused on data from one subgroup. Four studies from Europe and the Americas reported recurrence, with a combined prevalence of 11.4% and a 95% CI of 7.52%–16.94%, which was the highest among all complications. This suggested that recurrence is more prevalent in Europe and the Americas (see Figure S17).

#### 3.2.2 Prevalence of complications among women who underwent pelvic organ prolapse surgery without hysterectomy in different geographical locations

In patients with pelvic organs who have not undergone hysterectomy, the small number of studies may lead to a bias in conclusion; thus, we only focused on one subgroup. Four studies from the Americas, Europe, Australia and Asia reported overall complications, with a combined prevalence of 8.94% and a 95% CI of 2.46%–27.69%. the first highest prevalence of all symptoms (see Figure S18).

#### 3.2.3 Prevalence of complications among women with pelvic organ prolapse using different surgical methods

In POP patients undergoing hysterectomy, we analysed several studies to determine overall complications. The prevalence of overall complications was 11.01% (95% CI: 3.49%–29.94%) at laparoscopic surgery, 40.37% in one study in robotic surgery, 8.17% (95% CI: 2.15%–26.50%) in abdominal surgery and 8.39% (95% CI: 3.77%–17.51%) in vaginal surgery (see Figure S19). Laparoscopic, abdominal and vaginal surgery had relatively low overall complication rates, but this does not mean that they were without risk. Robotic surgery had a relatively high complication rate, but the data came from only one study, and thus it may have some limitations that need to be revalidated when more studies are undertaken.

In patients with POP who had not undergone hysterectomy, we also focused on overall complications. The pooled prevalence of overall complications was 7.80% (95% CI of 1.87%–27.49%) at laparoscopic surgery, 19.32% for abdominal surgery and 51.32% for vaginal surgery (see Figure S20). The prevalence of overall complications appears to be higher in abdominal and vaginal surgery than in laparoscopic surgery. However, as this came from a small number of studies, this could have prejudiced the figures , and further studies need to be undertaken.

The OR for recurrence between POP patients who had undergone hysterectomy and POP patients who had not undergone hysterectomy with laparoscopic surgery was 0.40 (95% CI: 0.24–0.67). The OR was 0.88 (95% CI: 0.43–1.80) for abdominal surgery and 2.01 (95% CI: 1.05–3.85) for vaginal surgery (see Figure S21).

### 3.3 Publication bias

Egger's test was used to determine publication bias. The *p* values were calculated based on Egger's test . As shown, studies reporting symptoms of overall complications have no significant bias (see Figures S24 and S25).

## 4 DISCUSSION

Outcomes following different surgical approaches were reported applying a predetermined set of criteria. Some studies focused on comparison of outcomes in‐between trial and control groups, depicting further applicability and failures themselves. In such situations, controlling extraneous variables could not be possible because of the unadjusted propensity across samples.^13^, ^14^, ^15^, ^16^

Some studies applied precautions prophylactically for achieving better post‐operative outcomes. It was observed that many studies were conducted retrospectively using clinical records of the study participants over a prolonged period of time, which limited the identification of some important outcomes. Although in prospective studies, most of the outcomes were limited to peri‐operative complications, 30‐day post‐operative complications, 90‐day readmission rates and 1‐year recurrence rates, they did provide short‐term and middle‐term outcomes that could be of clinical relevance.

Considering surgical approaches, the majority of studies focused on minimally invasive surgical approaches, including vaginal, laparoscopic, robotic or vaginally assisted laparoscopic approaches to surgery, rather than open abdominal route. Though the routes of surgery were often predetermined, it is important to note that one study reported that modification of the route of surgery could be achieved for those requiring a hysterectomy at the time of POP surgery by administering GnRh agonists preoperatively to debulk the uterine mass, thus facilitating a vaginal approach rather than an open abdominal approach.

The efficacy and success of different surgical approaches were evaluated using various study designs, such as retrospective cohort studies, RCTs and prospective observational studies. To evaluate efficacy and/or success, commonly measured outcomes of the majority of the studies reviewed were peri‐operative complications, such as pelvic organ damage, estimated blood loss, blood transfusions and post‐operative assessments of duration of hospital stay, operating time and 30‐day post‐operative complications (such as infections, readmission, re‐operative and recurrence rates, mesh‐associated pain and discomfort, and other quality of life issues affecting women). In POP surgery, the vaginal route was the most preferred minimally invasive surgical approach with less post‐operative complications. Despite this, 18 studies showed preference for laparoscopic surgery ^18‐20^. The studies intimated that this approach was the most effective technique for performing hysterectomies, in those with POP, among women older than 65 years of age and with high BMI (obese women), with or without mesh augmentation. This could simply be due to a surgeon's preference rather than better clinical outcomes. Robotic‐assisted approaches such as mesh‐augmented sacrocolpopexies demonstrated no difference in operating time and peri‐operative and post‐operative complications when compared with laparoscopic approaches.

Performing hysterectomy at the time of POP surgery is common, but over the last two decades, uterine preservation has become *de rigeur*. This was often in association with mesh augmentation to treat the prolapse. Although POP surgery has its associated complications, as discussed above, hysterectomy as a stand‐alone procedure has inherent risks and complications. Surgical approaches remain as discussed previously, but the type of hysterectomy (total or sub‐total, with or without removal of the fallopian tubes and/or ovaries) can have a bearing on outcomes.These include complications related directly to surgery such as excessive bleeding requiring blood transfusion, moderate bleeding without the necessity for a transfusion, surgical site infection, urinary tract infection, kidney infections and organ rupture such as perforated bowel. There are also indirect effects of the surgery, such as mental health impact due to low mood, anxiety, depression and cognitive disturbance. Both direct and indirect complications were identified among the patient groups studied.

The majority (*n* = 29) of studies on POP surgery identified post‐operative infections, recurrence rates and re‐operation rates as an outcome of the intervention applied. However, there were many extraneous factors associated with the subjects studied. These included varying surgical techniques, experience of performing surgeons, different physical and physiological characteristics of the sample studied, missing data in retrospective studies, prophylactic antibiotic usage and risks associated with study subjects. Some studies demonstrated vague outcome measures, and therefore, success or efficacy had to be evaluated carefully when attempting to compare different surgical techniques and several types of POP operations. Considering surgical techniques, the majority of the POP surgery studies considered total hysterectomies rather than partial or radical hysterectomies. However, the decision depended on the diagnosis and the patient's clinical picture. For example, in those having POP surgery and in whom there was an associated risk of ovarian cancer, prophylactic salpingectomies were performed.

Surgical techniques employed also varied depending on the symptomatology of the POP, severity of the disease, availability of suitable facilities and instruments, available best practice approaches and research evidence.

Although most studies focused on short‐term outcomes, some studies (*n* = 10) identified middle‐term and long‐term outcomes and associated factors of those outcomes. Significantly, recurrence and/or re‐operation rates following failures in POP surgical interventions or mesh‐associated pain and discomfort following mesh‐augmented POP surgery have resulted in further medical and surgical interventions. Mesh‐augmented vaginal prolapse surgery is now uncommon in the United Kingdom, following the publication of the NICE (National Institute for Health and Care Excellence) guidelines' recommendations in 2019, where mesh insertion was deemed to be only for research purposes and not for general widespread use. This was further supported by the widespread moratorium on mesh in all POP surgery by the Cumberlege Report *First Do No Harm* in 2020. It has also impacted the uptake of mesh‐augmented abdominal prolapse surgery (using laparoscopic, robotic or open approaches), though surgeons still accept it to continue to use mesh in such cases.^21^ However, increasingly, patients are not keen to have mesh inserted for any reason, and thus, in years to come, the use of laparoscopic ^18‐20^ and robotic approaches to mesh‐augmented POP surgery may decline.

## 5 LIMITATIONS

It is vital to acknowledge the lack of uniformity with health and clinical outcome reporting in the studies. The lack of medical histories and comorbidities, as well as any previous surgeries, were another issue. Histories around normal deliveries, miscarriages and those that did not have children are vital to compare and contrast complications and outcomes for those with POP or vaginal prolapse. BMI and menopausal status were often not reported, despite this being a vital component clinically when assessing complications and associated quality of life. Based on the available evidence, POP appears to be a multifactorial yet poorly understood condition with a complex pathology and aetiology. The lack of data describing the psychosocial dysfunction and contextual factors that could provide valuable insight to healthcare professionals to improve health and clinical outcomes could be a barrier to improve both clinical and patient‐reported outcomes. Most studies took place within high‐income countries with a Caucasian population. Lack of low–middle‐income country participation and representation of all ethnicities and races within high‐income countries are a barrier to promoting health equality and equity. Methodological rigour, including optimal study design and statistical approaches, should be considered for future studies, including the use of validated questionnaires, longer follow‐up periods and quantifiable measures such as mean, standard deviation, interquartile range and model parameters using random‐effect or fixed‐effect models. The use of social media platforms and multilingual tools could improve representation of underserved populations when studies are conducted within high‐income countries.

## 6 CONCLUSION

It is evident that high‐quality evidence is lacking, and this is a limiting factor to better understand diagnostic and treatment methods for women with POP. The negative experiences of POP patients require support to significantly improve their quality of life and reduce the prevalence and severity of complications. Future studies would benefit from better study designs, clearer definitions for clinical and patient‐reported outcomes, long‐term follow‐up outcomes, representation of different healthcare systems and ethnicities, and the use of statistically significant sample sizes.

## AUTHOR CONTRIBUTIONS

Gayathri Delanerolle, Peter Phiri and Sohier Elneil conceptualized this manuscript. Jian Qing Shi, Tharanga Mudalige, Vindya Pathiraja, Shuqi Wu, Ruishu Zhang and Gayathri Delanerolle conducted the analysis. All authors critically appraised and commented on all versions of the manuscript. All authors read and approved the final manuscript.

## CONFLICT OF INTEREST STATEMENT

All authors report no conflict of interest. The views expressed are those of the authors and not necessarily those of the NHS, the National Institute for Health Research, the Department of Health and Social Care or the academic institutions.

## Supporting information


**Figure S1.** Forest plot showing the log odds of recurrence among POP women that have had a hysterectomy.
**Figure S2.** Forest plot showing the log odds of readmission among POP women that have had a hysterectomy**.**
**Figure S3.** Forest plot showing the log odds of reoperation among POP women that have had a hysterectomy**.**
**Figure S4.** Forest plot showing the log odds of overall complications (other than recurrence, readmission, reoperation) among POP women that have had a hysterectomy.
**Figure S5.** Forest plot showing the log odds of bladder injury among POP women that haven’t had hysterectomy.
**Figure S6.** Forest plot showing the log odds of infections among POP women that haven’t had a hysterectomy.
**Figure S7.** Forest plot showing the log odds of mesh complications among women who underwent pelvic organ prolapse surgery without hysterectomy.
**Figure S8.** Forest plot showing the log odds of recurrence among women who underwent pelvic organ prolapse surgery without hysterectomy.
**Figure S9.** Forest plot showing the log odds of readmission among women who underwent pelvic organ prolapse surgery without hysterectomy.
**Figure S10.** Forest plot showing the log odds of reoperation among women who underwent pelvic organ prolapse surgery without hysterectomy.
**Figure S11.** Forest plot showing the log odds of overall complications (other than recurrence, readmission, reoperation) among women who underwent pelvic organ prolapse surgery without hysterectomy.
**Figure S12.** Forest plot showing the odds ratio of mesh complications among women who underwent pelvic organ prolapse surgery with or without hysterectomy.
**Figure S13.** Forest plot showing the odds ratio of recurrence among women who underwent pelvic organ prolapse surgery without hysterectomy.
**Figure S14.** Forest plot showing the odds ratio of readmission among women who underwent pelvic organ prolapse surgery without hysterectomy.
**Figure S15.** Forest plot showing the odds ratio of reoperation among women who underwent pelvic organ prolapse surgery without hysterectomy.
**Figure S16.** Forest plot showing the odds ratio of overall complications (other than recurrence, readmission, reoperation) among women who underwent pelvic organ prolapse surgery with or without hysterectomy.
**Figure S17.**Funnel plots of recurrence among women who underwent pelvic organ prolapse surgery with hysterectomy within different geographical locations.
**Figure S18.** Funnel plots of overall complications among women who underwent pelvic organ prolapse surgery without hysterectomy within different geographical locations.
**Figure S19.**Funnel plots with log odds of overall complications among women who underwent pelvic organ prolapse surgery with hysterectomy using different surgical methods.
**Figure S20.** Funnel plots with log odds of overall complications among women who underwent pelvic organ prolapse surgery without hysterectomy using different surgical methods.
**Figure S21.** Funnel plots with odds ratio of readmission among women who underwent pelvic organ prolapse surgery with or without hysterectomy using different surgical methods.
**Figure S22.** Funnel plots of 7 symptoms (reported in more than 3 studies) among POP women that have had a hysterectomy**.**
**Figure S23.** Funnel plots of 4 symptoms (reported in more than 3 studies) among POP women that haven’t had a hysterectomy.
**Figure S24.** P values for residual selection bias among POP women that have had a hysterectomy.
**Figure S25.** P values for residual selection bias among POP women that haven’t had a hysterectomy.
**Table S1.** Summarized results of sensitivity analysis among POP women that haven’t had a hysterectomy.
**Table S2.** Summarized results of sensitivity analysis among POP women that have had a hysterectomy.
**Table S1b.** Determination of the quality of studies included in the meta‐analysis (using The Newcastle‐Ottawa‐Scale).

## Data Availability

Not applicable.
