# Supplementary material for: Systematic review and meta‐analysis of the pelvic organ prolapse and vaginal prolapse among the global population
Source: BJUI Compass. 2024 Dec 10;6(1):e464. doi: 10.1002/bco2.464 (PMC11771496; doi:10.1002/bco2.464)
Supplement: Supplementary file 1 — Figure S1. Forest plot showing the log odds of recurrence among POP women that have had a hysterectomy. Figure S2. Forest plot showing the log odds of readmission among POP women that have had a hysterectomy. Figure S3. Forest plot showing the log odds of reoperation among POP women that have had a hysterectomy. Figure S4. Forest plot showing the log odds of overall complications (other than recurrence, readmission, reoperation) among POP women that have had a hysterectomy. Figure S5. Forest plot showing the log odds of bladder injury among POP women that haven’t had hysterectomy. Figure S6. Forest plot showing the log odds of infections among POP women that haven’t had a hysterectomy. Figure S7. Forest plot showing the log odds of mesh complications among women who underwent pelvic organ prolapse surgery without hysterectomy. Figure S8. Forest plot showing the log odds of recurrence among women who underwent pelvic organ prolapse surgery without hysterectomy. Figure S9. Forest plot showing the log odds of readmission among women who underwent pelvic organ prolapse surgery without hysterectomy. Figure S10. Forest plot showing the log odds of reoperation among women who underwent pelvic organ prolapse surgery without hysterectomy. Figure S11. Forest plot showing the log odds of overall complications (other than recurrence, readmission, reoperation) among women who underwent pelvic organ prolapse surgery without hysterectomy. Figure S12. Forest plot showing the odds ratio of mesh complications among women who underwent pelvic organ prolapse surgery with or without hysterectomy. Figure S13. Forest plot showing the odds ratio of recurrence among women who underwent pelvic organ prolapse surgery without hysterectomy. Figure S14. Forest plot showing the odds ratio of readmission among women who underwent pelvic organ prolapse surgery without hysterectomy. Figure S15. Forest plot showing the odds ratio of reoperation among women who underwent pelvic organ prolapse surgery [file BCO2-6-e464-s001.docx]

**Supplementary Material**

**Fig S1:** Forest plot showing the log odds of recurrence among PoP women that have had a hysterectomy.

**
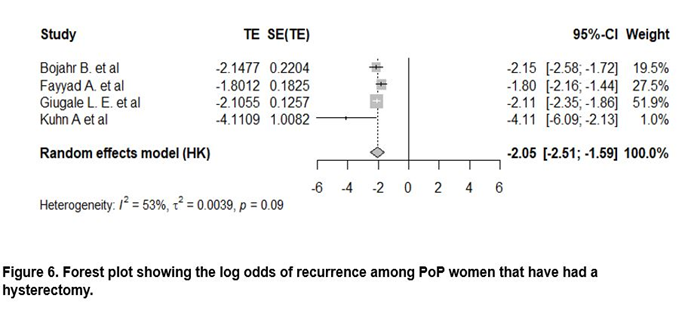
**

**Fig S2.** Forest plot showing the log odds of readmission among PoP women that have had a hysterectomy

**
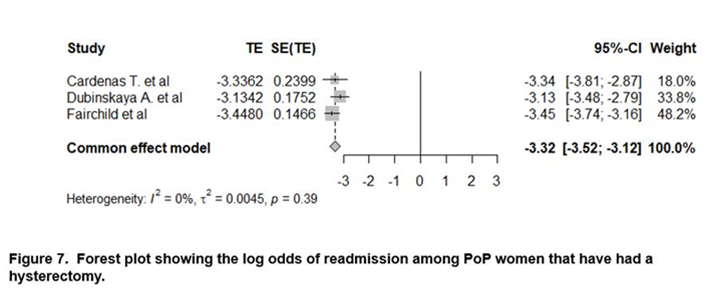
**

**Fig S3.** Forest plot showing the log odds of reoperation among PoP women that have had a hysterectomy.

**
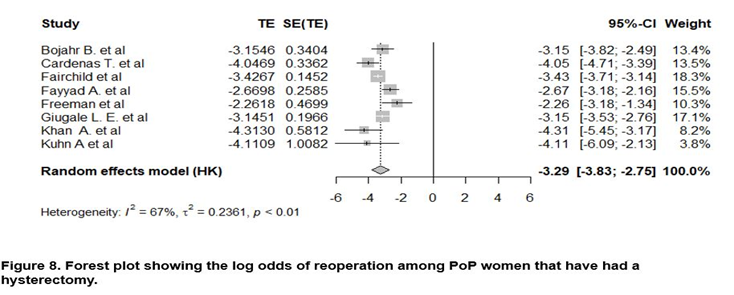
**

**Fig S4**. Forest plot showing the log odds of overall complications (other than recurrence, readmission, reoperation) among PoP women that have had a hysterectomy.

**
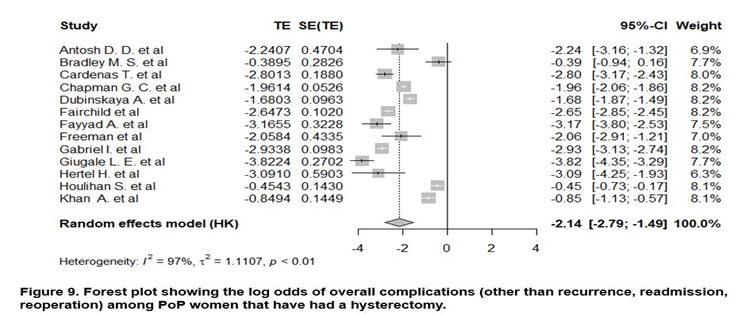
**

**Fig S5.** Forest plot showing the log odds of bladder injury among PoP women that haven’t

had hysterectomy.

**
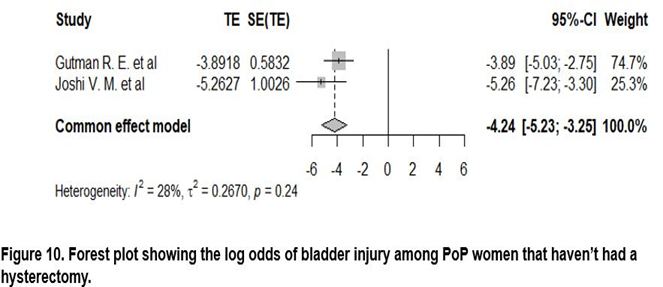
**

**Fig S6.** Forest plot showing the log odds of infections among PoP women that haven’t had a hysterectomy.

**
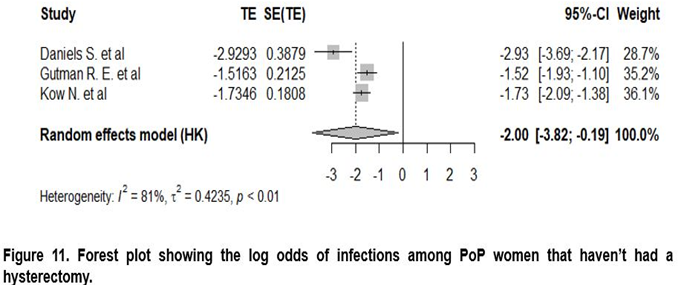
**

**Fig S7.** Forest plot showing the log odds of mesh complications among women who underwent pelvic organ prolapse surgery without hysterectomy.

**
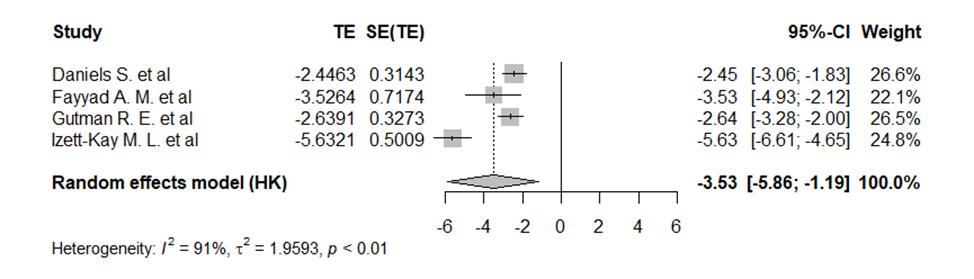
**

**Fig S8.** Forest plot showing the log odds of recurrence among women who underwent pelvic organ prolapse surgery without hysterectomy.

**
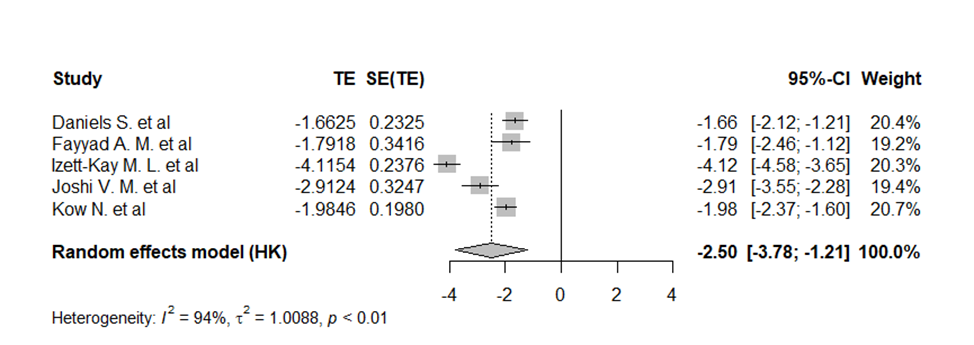
**

**Fig S9.** Forest plot showing the log odds of readmission among women who underwent pelvic organ prolapse surgery without hysterectomy.

**
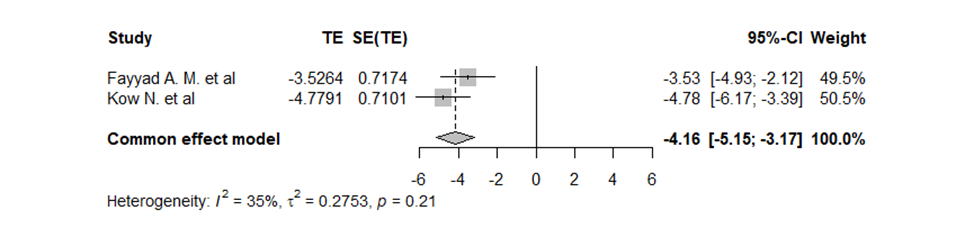
**

**Fig S10.** Forest plot showing the log odds of reoperation among women who underwent pelvic organ prolapse surgery without hysterectomy.

*
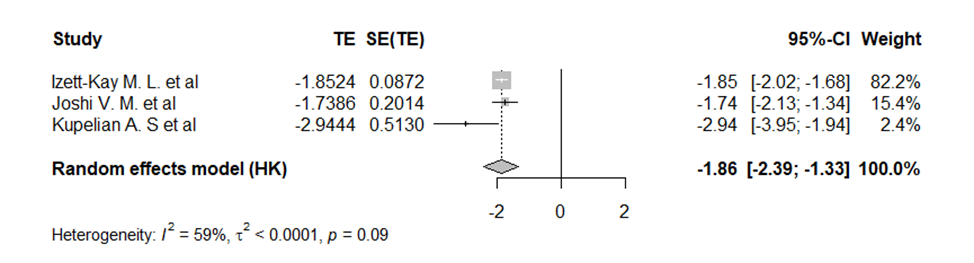
*

*Odds ratio of complications among women who underwent pelvic organ prolapse surgery with or without a hysterectomy*

The third meta-analysis reported the odds ratio of complications in women with pelvic organ prolapse that had a hysterectomy and those who had not. There were 6 studies, which focused on only mesh complications, recurrence of POP, readmission rates, reoperation rates, and overall complications.

The odds ratio for mesh complications between POP patients who had undergone hysterectomy and those who had not was 2.06 (95%CI: 0.48 to 8.89). The p-value of 0.99 indicates no statistical significance, while the heterogeneity of 0% suggests no significant variation in effect sizes across the studies (see Fig S12). However, it is important to note that the number of studies included in this analysis was limited, which may affect the reliability of the results.

The recurrence odds ratio comparing POP patients who had undergone hysterectomy with those who had not was 0.41 (95%CI: 0.09 to 1.89). The p-value of 0.45 indicates no significant difference between the two groups. The value of $I^{2}$=0% indicates minimal heterogeneity across the studies. However, further research is necessary to confirm these findings (see Fig S13) since there were only two studies in this meta-analysis.

The odds ratio for readmission between POP patients who had undergone hysterectomy and who had not was 1.07, with a 95%CI of (0.93 to 1.24). The observed p-value of 0.04 indicates a significant difference between the two groups. Moreover, the studies exhibit a moderate level of heterogeneity, indicated by a value of 70%. These findings highlight the significance of assessing the potential impact of hysterectomy on readmission rates among patients with pelvic organ prolapse (see Fig S14). The reoperation odds ratio comparing POP patients who had undergone hysterectomy with those who had not undergone hysterectomy was 1.05, with a 95%CI of 0.96 to 1.14. The statistical test revealed a p-value of 0.18, indicating significant differences between the two groups ($I^{2}$= 41%) (refer to Fig S15).

The odds ratio for POP patients who had undergone hysterectomy and who had not undergone hysterectomy of overall complications (excluding recurrence, readmission, and reoperation) was 1.03 (95%CI: 0.46 to 2.30). Non-significant difference existed between the two groups (p = 0.39), and the heterogeneity among the studies was relatively low ($I^{2}$= 0%). Further studies are required to validate these findings (see Fig S16).

Hysterectomy, in women with POP, was associated with an increased risk of recurrence of POP, readmission rates, reoperation rates, and overall complications. However, these increased risks were not statistically significant. This may be due to the limited number of studies included in the analysis, or the differences in study design, patient populations, and some other potential confounding factors. Future studies are needed to explore these issues in further depth and provide stronger evidence to support clinical practice.

**Fig S11.** Forest plot showing the log odds of overall complications (other than recurrence, readmission, reoperation) among women who underwent pelvic organ prolapse surgery without hysterectomy.

*
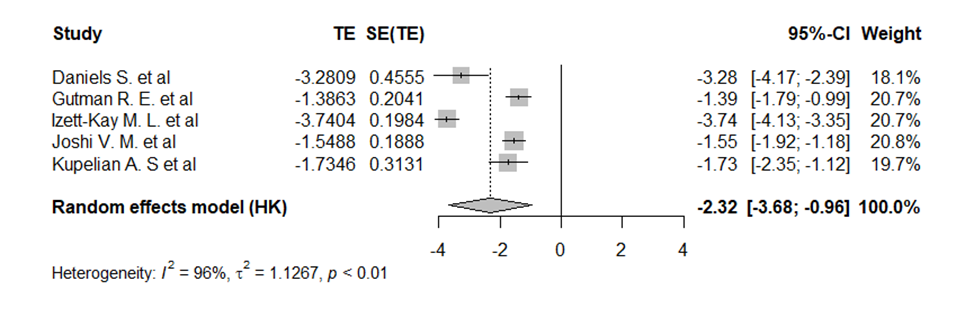
*

**Fig S12.** Forest plot showing the odds ratio of mesh complications among women who underwent pelvic organ prolapse surgery with or without hysterectomy.

*
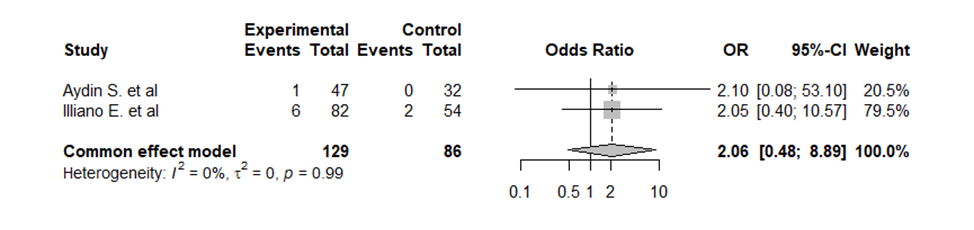
*

**Fig S13.** Forest plot showing the odds ratio of recurrence among women who underwent pelvic organ prolapse surgery without hysterectomy

*
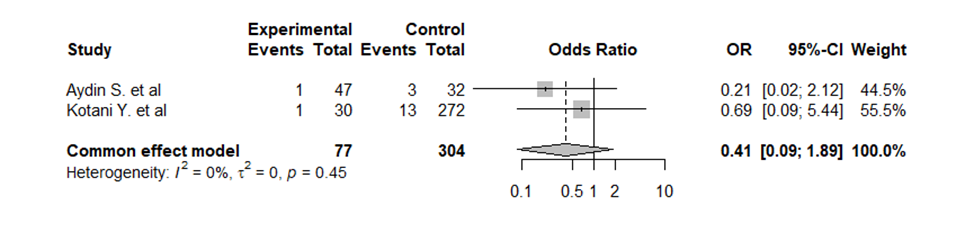
*

**Fig S14** Forest plot showing the odds ratio of readmission among women who underwent pelvic organ prolapse surgery without hysterectomy.

*
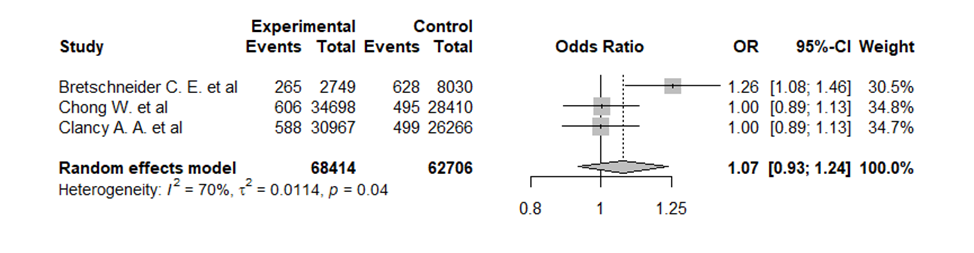
*

**Fig S15.** Forest plot showing the odds ratio of reoperation among women who underwent pelvic organ prolapse surgery without hysterectomy.

*
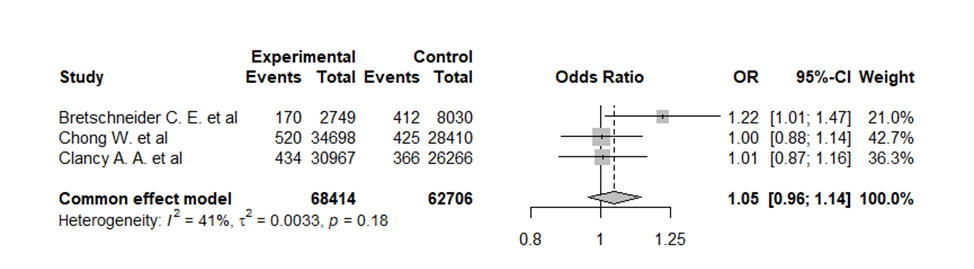
*

**Fig S16.** Forest plot showing the odds ratio of overall complications (other than recurrence, readmission, reoperation) among women who underwent pelvic organ prolapse surgery with or without hysterectomy.

*
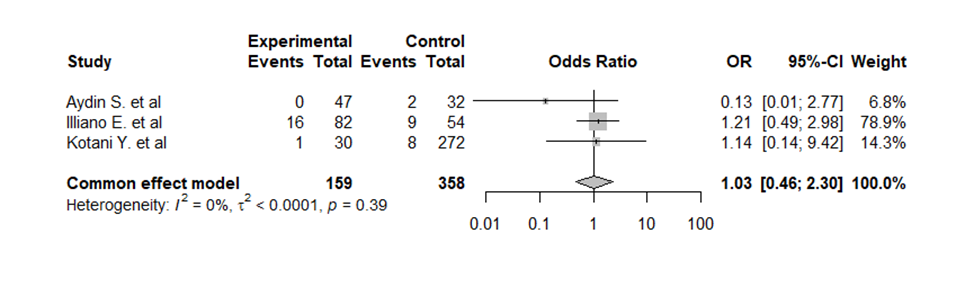
*

**Fig S17.**Funnel plots of recurrence among women who underwent pelvic organ prolapse surgery with hysterectomy within different geographical locations.


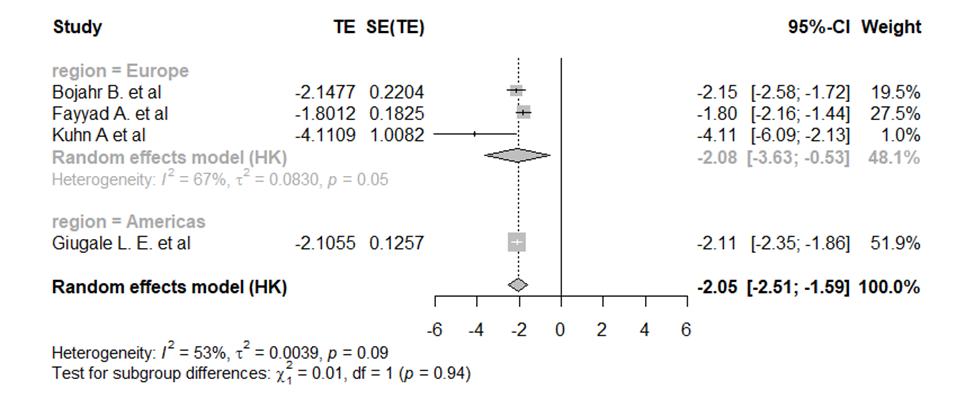


**Fig S18.** Funnel plots of overall complications among women who underwent pelvic organ prolapse surgery without hysterectomy within different geographical locations.


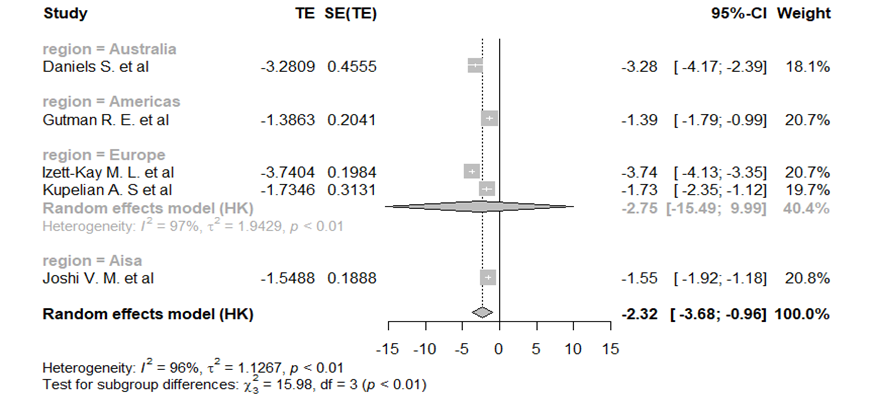


**Fig S19.**Funnel plots with log odds of overall complications among women who underwent pelvic organ prolapse surgery with hysterectomy using different surgical methods.

*
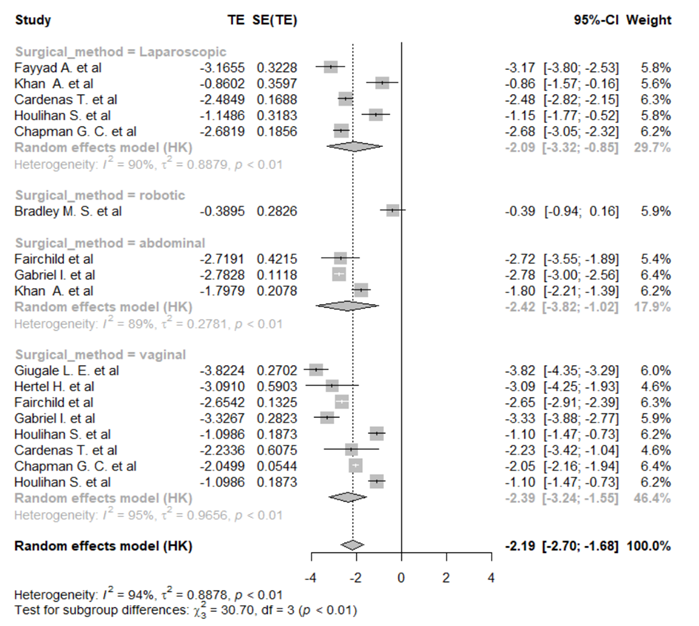
*

**Fig S20.** Funnel plots with log odds of overall complications among women who underwent pelvic organ prolapse surgery without hysterectomy using different surgical methods.

*
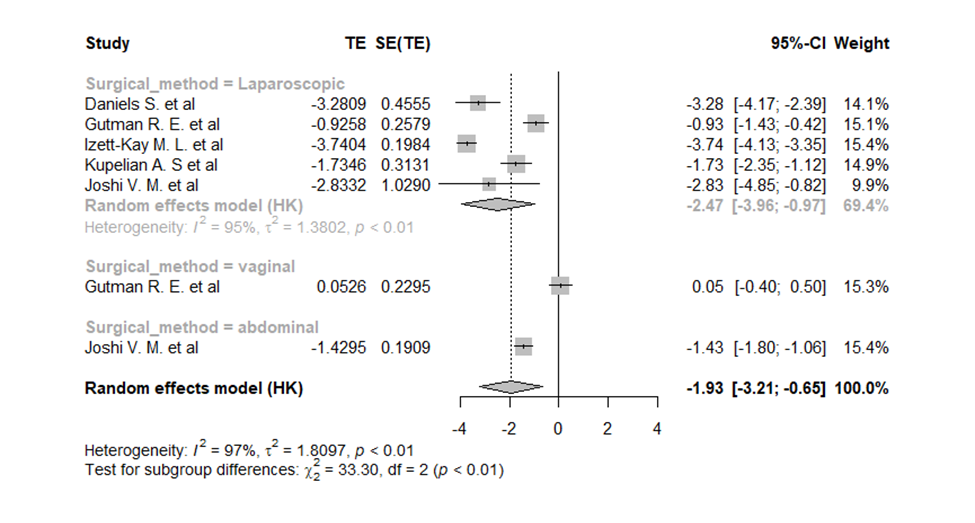
*

**Fig S21.**Funnel plots with odds ratio of readmission among women who underwent pelvic organ prolapse surgery with or without hysterectomy using different surgical methods.

*
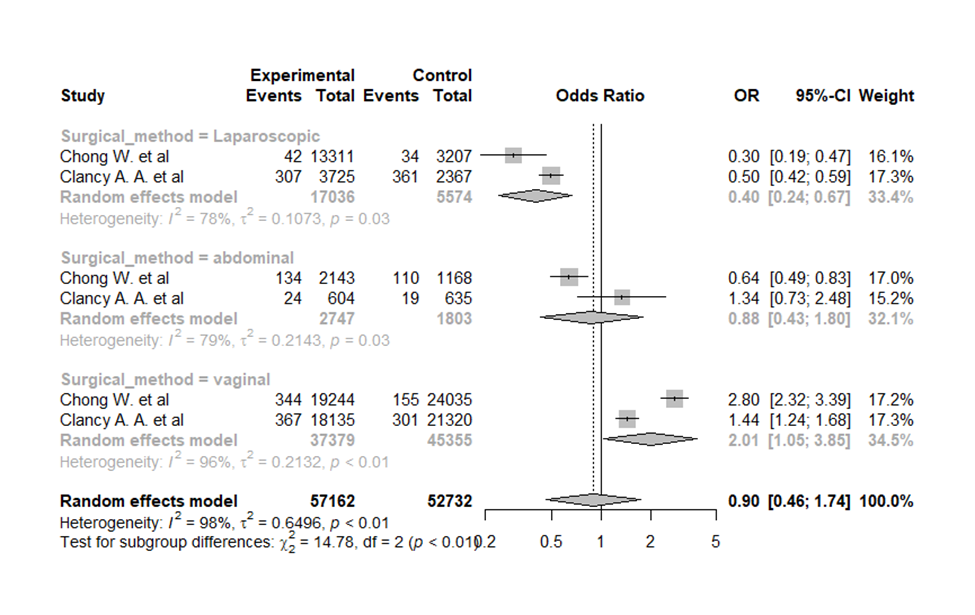
*

*Sensitivity analysis*

Many studies were outside the 95%CI as demonstrated within the funnel plots. Therefore, a sensitivity analysis was conducted using a Copas selection model ^56 57^ to adjust the pooled prevalence (Table S1 and S2) to derive tangible conclusions and better understand the reliability of the study findings.

In Table S1 and S2, the proportion of selected studies varied, and the changes in the P value of the residual selection bias are depicted in Fig S24-S25. The Copas model (CSM) was used to determine bias within studies based on P values exceeding 0.1. The proportion of studies used within the CSM are listed within Table S1 and S2. It is evident the CSM selected 40.73% studies with infections as a symptom among women who underwent pelvic organ prolapse surgery without hysterectomy, while the remaining 59.27% indicated a significant standard error, demonstrating inadequate quality and high heterogeneity, thus were excluded. The CSM selected 69.73% of trials with recurrence as a symptom among pelvic organ prolapse women that have had a hysterectomy. The result from the CSM was compared to a random effects model (REM), indicating P values exceeding 0.05, which demonstrates a lack of statistical significance. Therefore, the results of this study are consistent and provide robust conclusions.

*Prevalence of complications,*

Funnel plots present complications (Fig S22-S23) indicating high heterogeneity. Many studies were outside the scope of 95%CI which made it challenging to intuitively detect the bias.

1. Bladder injury (2) Infections

*
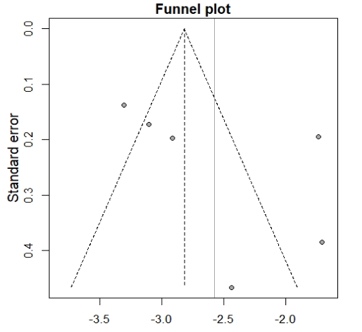

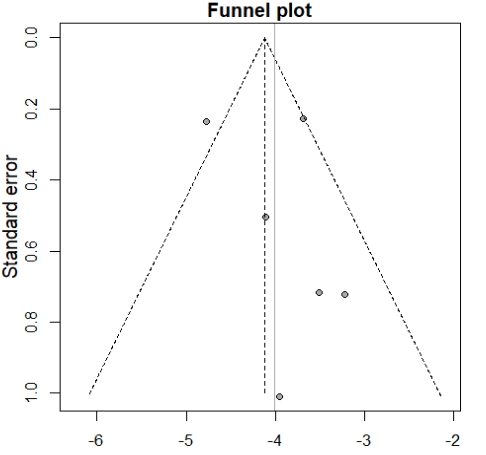
*

*
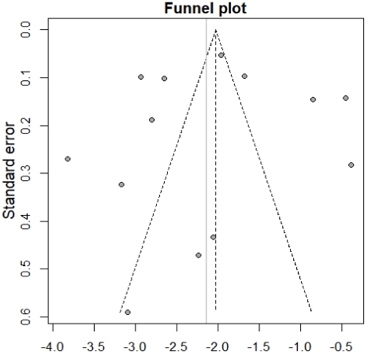
*(3)Mesh (4)Overall complications

*
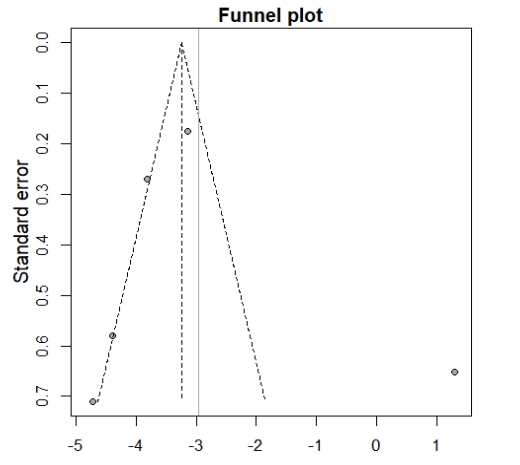
*

*
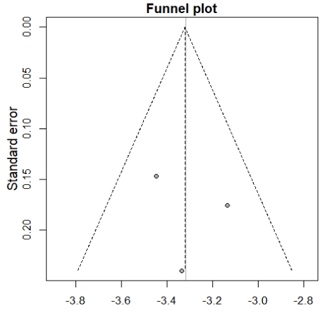
*(5) Readmission (6) Recurrence

*
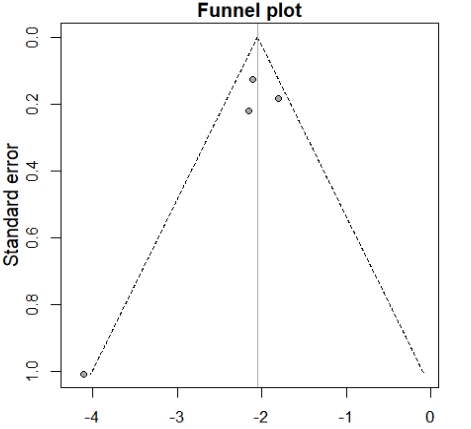
*

(7)Reoperation

*
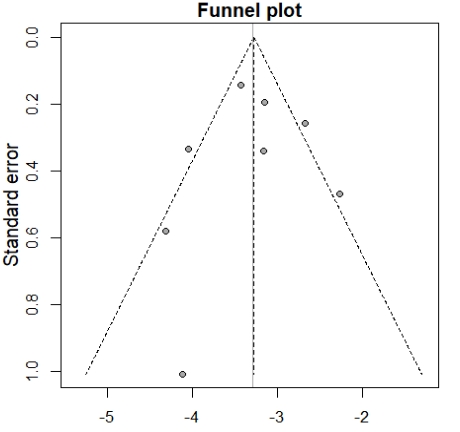
*

**Fig S22.**Funnel plots of 7 symptoms (reported in more than 3 studies) among PoP women that have had a hysterectomy.

(1)Infections (2)Overall complications

*
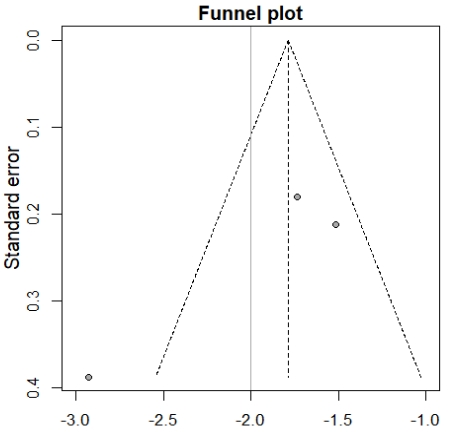

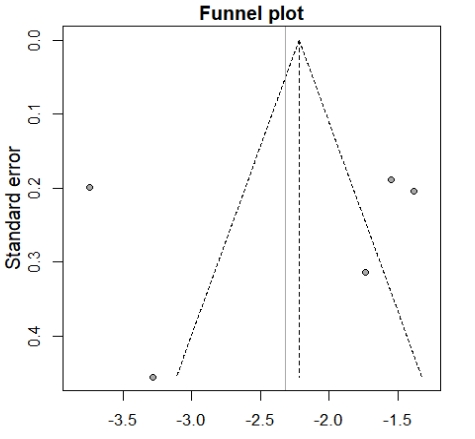
*

(3)Recurrence (4)Reoperation

*
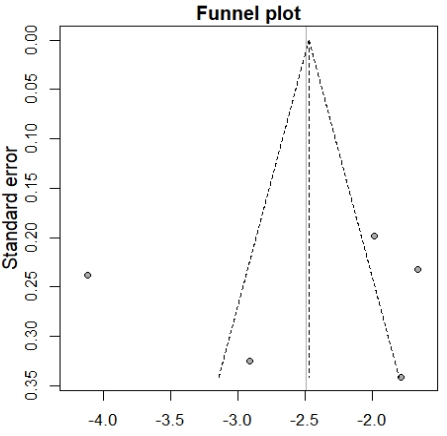

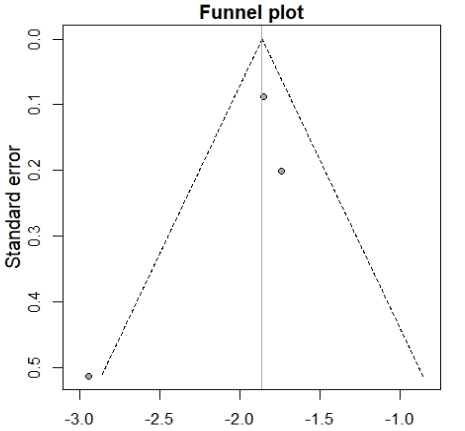
*

**Fig S23.**Funnel plots of 4 symptoms (reported in more than 3 studies) among PoP women that haven’t had a hysterectomy.

*Publication Bias - Egger’s test*

The proportion of selected studies varied, and the changes in the P-value of the residual selection bias are depicted below in Fig S24-S25. Egger’s test was used to determine publication bias.

(1)Bladder injury (2) Infections

*
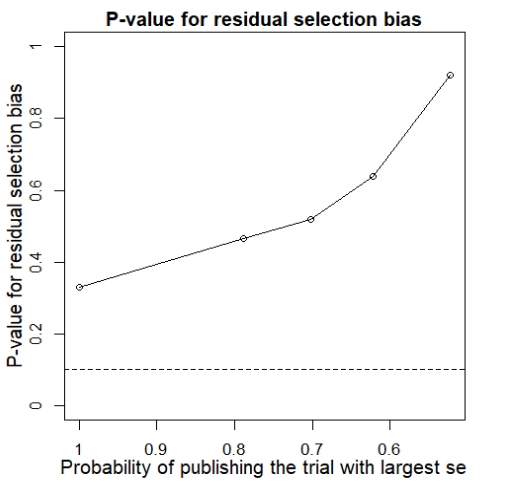

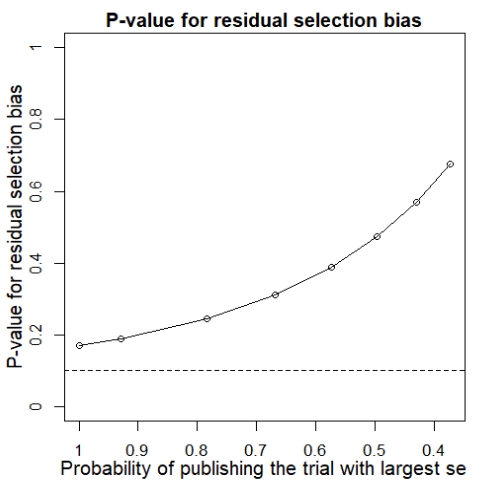
*

(3)Mesh (4)Overall complications

*
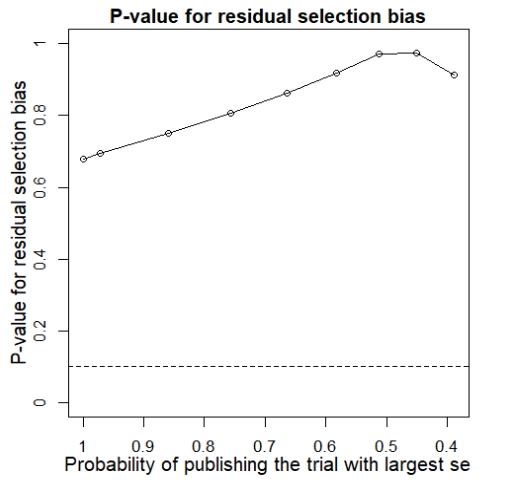

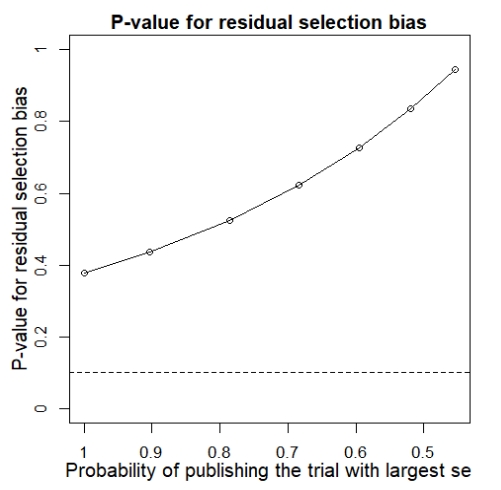
*

(5) Readmission (6)Recurrence

*
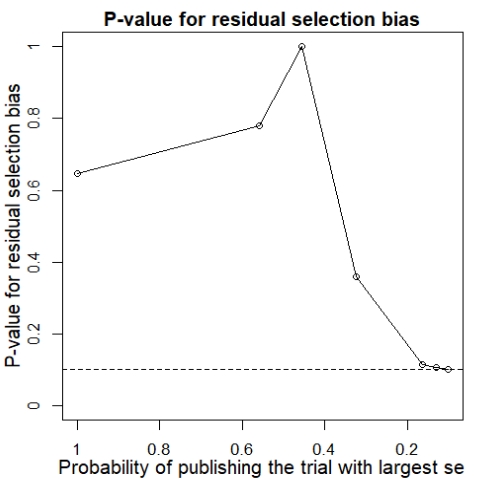

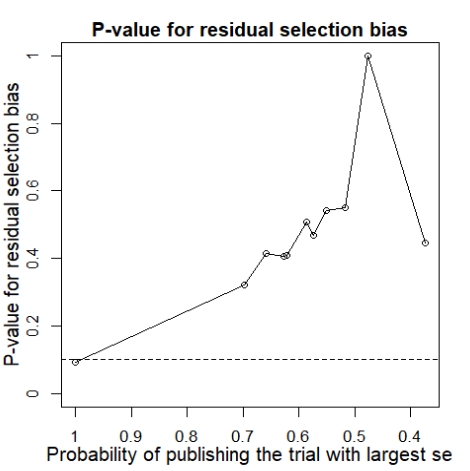
*

(7)Reoperation


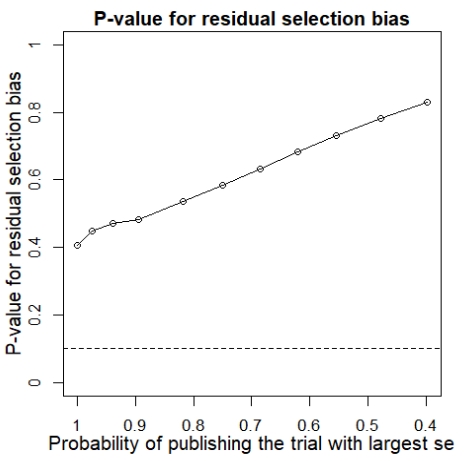


**Fig S24.**P values for residual selection bias among PoP women that have had a hysterectomy.

1. Infections (2)Overall complications

*
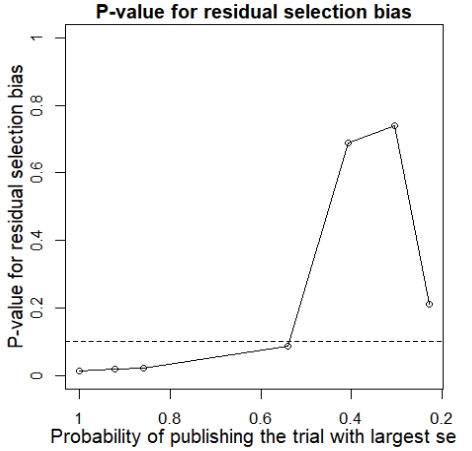

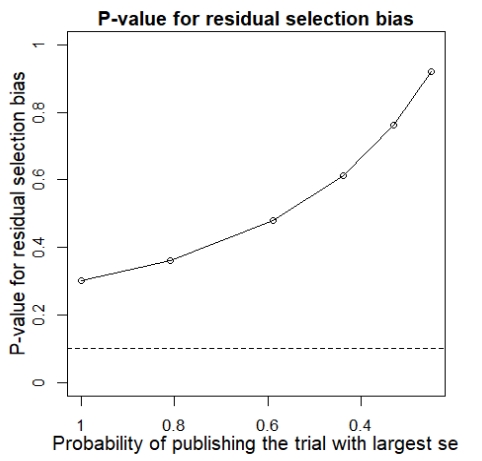
*

(3)Recurrence (4)Reoperation

**
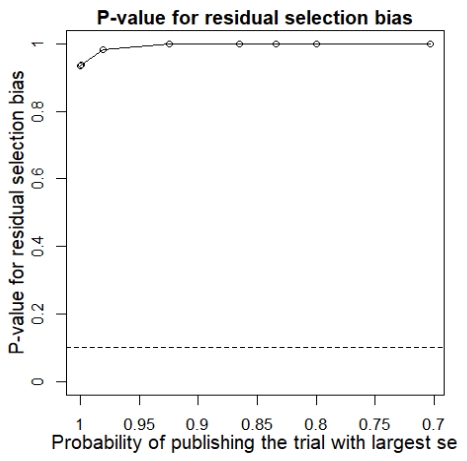

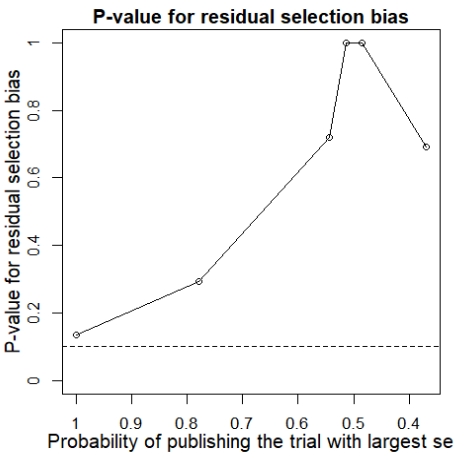
**

**Fig S25.**P values for residual selection bias among PoP women that haven’t had a hysterectomy.

**Table S1 Summarized results of sensitivity analysis among PoP women that haven’t had a hysterectomy.**

| **Phenotype** | **N of study** | **Model** | **Probability of publishing study with largest standard error** | **Proportion(%)** | **Lower(%)** | **Upper(%)** | **p value for differences between two conclusions** |
| --- | --- | --- | --- | --- | --- | --- | --- |
| Infections | 3 | Copas selection model | 40.73% | 16.79% | 10.96% | 24.87% | 0.6898 |
| Recurrence | 5 | Copas selection model | 100% | 7.62% | 3.51% | 15.73% | 0.9362 |
| Reoperation | 3 | Copas selection model | 100% | 13.46% | 11.76% | 15.37% | 0.1346 |
| Overall complications | 5 | Copas selection model | 100% | 8.99% | 3.99% | 18.97% | 0.4653 |

**Table S2 Summarized results of sensitivity analysis among PoP women that have had a hysterectomy.**

| **Phenotype** | **N of study** | **Model** | **Probability of publishing study with largest standard error** | **Proportion** | **Lower** | **Upper** | **p value for differences between two conclusions** |
| --- | --- | --- | --- | --- | --- | --- | --- |
| Bladder injury | 6 | Copas selection model | 100% | 1.76% | 1.05% | 2.93% | 0.3299 |
| Infections | 6 | Copas selection model | 100% | 7.05% | 4.29% | 11.36% | 0.1706 |
| Mesh | 5 | Copas selection model | 100% | 4.87% | 0.79% | 24.81% | 0.6780 |
| Recurrence | 4 | Copas selection model | 69.73% | 11.50% | 9.77% | 13.49% | 0.3214 |
| Readmission | 3 | Copas selection model | 100% | 3.48% | 2.87% | 4.22% | 0.6479 |
| Reoperation | 8 | Copas selection model | 100% | 3.63% | 2.51% | 5.23% | 0.4065 |
| Overall complications | 10 | Copas selection model | 100% | 10.54% | 6.25% | 17.25% | 0.3775 |

**Table S1b: Determination of the quality of studies included in the meta-analysis (using The Newcastle-Ottawa-Scale)**

| **No** | **Authors** | **Selection (S)** | | | | **Comparability (C)** | **Exposure/**  **Outcome (E/O)** | | | **Total Stars** | **S** | **C** | **E/O** | **Conclusion** |
| --- | --- | --- | --- | --- | --- | --- | --- | --- | --- | --- | --- | --- | --- | --- |
|  |  | 1 | 2 | 3 | 4 | 1 | 1 | 2 | 3 |  |  |  |  |  |
| 1 | Abd Elzaher M. *et al* | * | * |  | * | * |  | * | * | ****** | Good | Good | Good | Good |
| 2 | Antosh D. D. *et al* | * |  |  | * | * | * | * |  | ***** | Fair | Good | Good | Fair |
| 3 | Arsene E. *et al* | * |  | * | * | * | * | * |  | ****** | Good | Good | Good | Good |
| 4 | Aydin S. *et al* | * |  | * | * | * | * | * | * | ******* | Good | Good | Good | Good |
| 5 | Barboglio P.G. *et al* | * |  | * |  | * | * | * |  | ***** | Fair | Good | Good | Fair |
| 6 | Bogani G. *et al* | * |  | * | * | * | * | * |  | ****** | Good | Good | Good | Good |
| 7 | Bogani G. *et al* | * |  | * | * | * | * | * |  | ****** | Good | Good | Good | Good |
| 8 | Bojahr B. *et al* | * |  | * | * | * | * | * |  | ****** | Good | Good | Good | Good |
| 9 | Bradley M. S. *et al* | * |  | * | * | * | * | * | * | ******* | Good | Good | Good | Good |
| 10 | Bretschneider C. E. *et al* | * |  | * | * | * | * | * |  | ****** | Good | Good | Good | Good |
| 11 | Bui C. *et al* |  |  |  |  |  |  |  |  |  |  |  |  |  |
| 12 | Cardenas T. *et al* | * |  | * |  | * | * | * |  | ***** | Fair | Good | Good | Fair |
| 13 | Ceccaroni M. *et al* | * |  | * | * | * | * | * |  | ****** | Good | Good | Good | Good |
| 14 | Cengiz H. *et al* | * | * |  |  | * | * | * |  | ***** | Fair | Good | Good | Fair |
| 15 | Chapman G. C. *et al* | * |  | * | * | * | * | * |  | ****** | Good | Good | Good | Good |
| 16 | Chong W. *et al* | * |  | * | * | * | * | * |  | ****** | Good | Good | Good | Good |
| 17 | Clancy A. A. *et al* | * |  | * | * | * | * | * |  | ****** | Good | Good | Good | Good |
| 18 | Costantini E. *et al* | * |  | * | * | * | * | * |  | ****** | Good | Good | Good | Good |
| 19 | Crouss T. *et al* | * |  | * | * | * | * | * |  | ****** | Good | Good | Good | Good |
| 20 | Dallas K. *et al* |  |  |  |  |  |  |  |  |  |  |  |  |  |
| 21 | Daniels S. *et al* | * |  | * | * | * | * | * | * | ******* | Good | Good | Good | Good |
| 22 | Dubinskaya A. *et al* | * |  | * | * | * | * | * | * | ******* | Good | Good | Good | Good |
| 23 | Fairchild *et al* | * |  | * | * | * | * | * |  | ****** | Good | Good | Good | Good |
| 24 | Fayyad A. *et al* | * |  | * | * | * | * | * | * | ******* | Good | Good | Good | Good |
| 25 | Fayyad A. M. *et al* |  |  |  |  |  |  |  |  |  |  |  |  |  |
| 26 | Fernandez H. *et al* | * |  | * | * | * | * | * |  | ****** | Good | Good | Good | Good |
| 27 | Ferreira H. *et al* | * |  | * | * | * | * | * | * | ******* | Good | Good | Good | Good |
| 28 | Freeman *et al* | * | * | * | * | * | * | * | * | ******** | Good | Good | Good | Good |
| 29 | Gabriel I. *et al* | * |  | * | * | * | * | * | * | ******* | Good | Good | Good | Good |
| 30 | Gagnon L. H. *et al* | * |  | * | * | * | * | * |  | ****** | Good | Good | Good | Good |
| 31 | Giugale L. E. *et al* | * |  | * | * | * | * | * | * | ******* | Good | Good | Good | Good |
| 32 | Gutman R. E. *et al* | * |  | * | * | * | * | * | * | ******* | Good | Good | Good | Good |
| 33 | Hertel H. *et al* | * |  | * | * | * | * | * | * | ******* | Good | Good | Good | Good |
| 34 | Houlihan S. *et al* | * |  | * | * | * | * | * |  | ****** | Good | Good | Good | Good |
| 35 | Illiano E. *et al* | * |  | * | * | * | * | * | * | ******* | Good | Good | Good | Good |
| 36 | Izett-Kay M. L. *et al* |  |  |  |  |  |  |  |  |  |  |  |  |  |
| 37 | Joshi V. M. *et al* |  |  |  |  |  |  |  |  |  |  |  |  |  |
| 38 | Jugnet N. *et al* | * |  | * | * | * | * | * |  | ****** | Good | Good | Good | Good |
| 39 | Khan A. *et al* | * |  | * | * | * | * | * |  | ****** | Good | Good | Good | Good |
| 40 | Kotani Y. *et al* | * |  | * | * | * | * | * |  | ****** | Good | Good | Good | Good |
| 41 | Kow N. *et al* | * |  | * | * | * | * | * | * | ******* | Good | Good | Good | Good |
| 42 | Kuhn A *et al* | * |  | * | * | * | * | * |  | ****** | Good | Good | Good | Good |
| 43 | Kupelian A. S *et al* | * |  | * | * | * | * | * | * | ******* | Good | Good | Good | Good |
| 44 | Lauretta A *et al* | * |  | * | * | * | * | * | * | ******* | Good | Good | Good | Good |
